# Editorial: Trends in Comparative Endocrinology and Neurobiology

**DOI:** 10.3389/fendo.2017.00338

**Published:** 2017-12-01

**Authors:** Hubert Vaudry, Olivier Kah

**Affiliations:** ^1^Université de Rouen, Mont-Saint-Aignan, France; ^2^University of Rennes 1, Rennes, France

**Keywords:** peptide hormones and neuropeptides, biological rhythms, reproduction, endocrine disrupters, behavior

**Editorial on the Research Topic**

**Trends in Comparative Endocrinology and Neurobiology**

Neil Campbell once described evolution as the “overarching theme of biology.” Indeed, many comparative studies have significantly contributed to major discoveries in biological sciences. This is particularly true in the field of comparative endocrinology and neurobiology. For instance, the concept of neurosecretion has been established from studies conducted in invertebrates and fish (1, 2). Similarly, a number of peptide hormones and neuropeptides [e.g., melanin-concentrating hormone, urocortin, urotensin II (UII), glucagon-like peptide-1, stanniocalcin, etc.] have been identified in non-mammalian vertebrates before being “re-discovered” in mammals including humans (3–12). These breakthroughs bear witness to the power of the comparative approach to the current knowledge in endocrinology and neurobiology. They certainly support the famous assertion by Theodosius Dobzhansky that “Nothing in biology makes sense except in the light of evolution” (13).

This research topic is a compilation of contributions stemming from the 27th Conference of the European Comparative Endocrinologists (CECE2014) held in Rennes, France, that illustrates various facets of current comparative endocrinology and neurobiology investigations.

The neuropeptide allatotropin (AT), which was originally isolated in the central nervous system of the tobacco hornworm, was named after its ability to stimulate juvenile hormone (JH) biosynthesis from the corpora allata. Lismont et al. have characterized the cDNA sequence of AT and its receptor in the desert locust *Schistocerca gregaria*. Expression of the receptor cDNA in a mammalian cell line reveals that AT causes Ca^2+^ mobilization and stimulates cAMP production. On locust isolated organs, AT stimulates gut contraction and JH biosynthesis.

Juvenile hormone is an ester of farnesol, a cholesterol precursor found in all eukaryotes. Since, in insects, JH maintains larvae in a juvenile state, De Loof et al. raise the provocative question as to whether farnesol-like endogenous compounds act as anti-aging factors in vertebrates. Indeed, tissue extracts from mammalian organs do display JH activity in insects. In addition, farnesol, its precursor, and/or its metabolites occur in various mouse tissues. Thus, the authors elaborate a hypothesis on the possible roles of these compounds in vertebrates.

A large proportion of regulatory peptides are α-amidated. SALFamides are a family of myorelaxant neuropeptides that occur in echinoderms. Elphick et al. have characterized the SALFamide precursors in representative species of the five echinoderm classes. From these data, they can propose different scenarios regarding the phylogenetic history of the SALFamide neuropeptide family.

The neurohypophysial nonapeptides arginine vasopressin (AVP) and oxytocin are the mammalian representatives of a large family of neuropeptides that appeared very early during evolution. Banerjee et al. have cloned the cDNA encoding the precursors of vasotocin and isotocin, the AVP, and oxytocin counterparts, in two air-breathing catfish. The two precursor genes are expressed both in the brain and the follicular envelope of the ovary and display higher expression during the reproductive season, suggesting a possible role in the control of ovarian activity.

Three families of G protein-coupled receptors (GPCRs) have been identified in mammals. There is now clear evidence that oligomerization of GPCRs impacts their trafficking, ligand binding, and signaling. While intrafamily oligomer formation has been extensively studied, interfamily oligomerization is less documented. In their review, Ng and Chow describe the different methods applied to study oligomerization of GPCRs and summarize the current knowledge concerning interfamily GPCR heteromerization.

Parathyroid hormone (PTH) is a peptide that plays a crucial role in the regulation of calcium homeostasis. PTH belongs to a family of peptides that includes PTH-related peptide and the tuberoinfundibular peptide of 39 residues (TIP39 also called PTH2). On et al. describe the molecular evolution of these peptides and their receptors in vertebrates. Their review indicates that these peptide–receptor systems appeared early during evolution, possibly in invertebrates.

In mammals, the timing of ovulation is crucial for the success of reproduction: plasma estradiol level acts as an indicator of oocyte maturation while peptidergic neurons within the suprachiasmatic nucleus signal the time of the day and synchronize female fertility with the period of maximal activity and sexual motivation. Simonneaux and Bahougne summarize the literature pertaining to the inhibitory and stimulatory actions of estradiol on the reproductive axis and describe the roles of AVP and vasoactive intestinal peptide in the timing of the luteinizing hormone (LH) surge. They also review the contribution of peripheral clocks within the reproductive organs in the timing of female reproduction. Finally, they discuss the impact of shift work on female fertility.

In photoperiodic mammals, the duration of melatonin secretion during the night regulates seasonal changes of physiological functions. Chakir et al. show that, in the Syrian hamster, nocturnal melatonin exerts chronomodulatory effects on cortisol, leptin, insulin, and glucose daily rhythms. Their study thus provides important information regarding the role of melatonin in the circadian control of energy metabolism.

Besides the pineal gland, melatonin is synthesized by several organs including the retina and gastrointestinal tract. Mukherjee and Maitra review the physiological significance of gut-derived melatonin in vertebrates. While pineal-derived melatonin acts mainly as a neurohormone/neuromodulator, gut melatonin appears to act primarily locally as an autocrine/paracrine factor.

Domestic animals are fruitful models for comparative endocrinologists and neurobiologists. In spring, ewes normally do not ovulate, but exposure to a ram or to ram odor can induce an LH surge and ovulation. This pheromone response, known as the “ram effect,” can be used to compact lambing in a herd. Fabre-Nys et al. describe the neuroanatomical pathways and neurochemical mechanisms underlying the ram effect. They also show that the ram effect is a relevant model in which to address more general questions regarding the neuronal and endocrine regulation of the hypothalamic–pituitary–gonadal axis.

Kisspeptin has recently emerged as a major player of the neuroendocrine control of reproduction and puberty. Skrapits et al. review the literature regarding the occurrence of various neurotransmitters and neuropeptides in kisspeptin neurons both in laboratory animals and in humans. Their data reveal that human kisspeptin neurons express far more neuropeptides than those of laboratory species, suggesting that the fine control of fertility differs substantially between primates and non-primates.

Age-related dysfunctions in the endocrine system are usually associated with a decline of cognitive functions and a higher risk of neurodegenerative diseases. Blair et al. review the evidence that changes in gonadal steroids, LH, and even sex hormone-binding globulin can be involved in learning and memory impairment and development of Alzheimer’s disease. These data could lead to novel therapeutic strategies for the treatment of neurodegenerative disorders.

Aromatase catalyzes the conversion of testosterone into 17β-estradiol, and the inactivation of 17β-estradiol into catechol estrogens. Charlier et al. summarize the data showing that aromatase activity can be rapidly modified through phosphorylation/dephosphorylation processes. The fine tuning of aromatase phosphorylation in specific brain areas may thus account for the multiple physiological and behavioral effects of estrogens.

The peptide hormones adrenocorticotropin and melanocyte-stimulating hormones act through five GPCRs collectively called melanocortin receptors. The functional expression of this family of receptors is regulated by two melanocortin receptor accessory proteins, MRAP1 and MRAP2. Jackson et al. elaborate on the impact of MRAP deletions in adrenal deficiency and obesity, as revealed by animal model studies and human mutations.

The neuropeptide UII is a potent vasoconstrictor. UII belongs to a family of peptides that also includes three UII-related peptides, i.e., URP, URP1, and URP2. Vanegas et al. have compared the central and peripheral actions of UII, URP1 and URP2 on cardioventilatory and locomotor functions in unanesthetized trout. They show that intracerebroventricular injection of each of the three neuropeptides increases ventilation, blood pressure, heart rate, and locomotion with different potencies, whereas intraarterial administration of only a high dose of UII and URP1 (but not URP2) stimulates ventilation and locomotor activity.

In invertebrates, as in vertebrates, serotonin (5-HT) exerts multiple behavioral and neurophysiological actions. In the insect *Rhodnius proxilus*, the chief Chagas disease vector, 5-HT acts as a diuretic hormone that regulates hemolymph osmolarity after its dramatic feeding bout. Paluzzi et al. have cloned the 5-HT2b receptor cDNA in *R. proxilus* and studied its pharmacological profile on transfected cells and isolated organs.

In mammals, transthyretin (TTR) acts as a thyroxine (T4)-binding protein whereas, in sub-mammalian vertebrates, TTR binds preferably the active form of thyroid hormones, triiodothyronine (T3). Richardson reviews the evolution of the TTR gene and describes how subtle changes in the protein structure result in the switch of the ligand from T3 to T4.

The glycoprotein hormone (GPH) family encompasses LH, follicle-stimulating hormone, thyroid-stimulating hormone, and chorionic gonadotropin. Although GPHs are present only in vertebrates, related GPH subunit ancestor genes have been identified in most vertebrates phyla. Cahoreau et al. provide an extensive and critical look at the structure and function of GPHs and their receptors.

5′AMP-activated protein kinase (AMPK) is a protein kinase that is activated by ATP deficiency. AMPK is primarily expressed in the liver and muscle where it acts as an energy sensor. In invertebrates and vertebrates, AMPK is also expressed in the gonads. Bertoldo et al. take a broad comparative view to examine the role of AMPK in the interplay between the regulation of energy homeostasis and reproductive functions.

Steroid hormones influence the fertility, development, and survival of parasites; reciprocally, parasite infection often affects plasma steroid levels in the host. Romano et al. provide evidence that a number of parasites express several steroidogenic enzymes and can synthesize various steroid hormones including ecdysteroids, sex steroids, and corticosteroids.

Endocrine-disrupting chemicals (EDCs) are pharmaceutical or environmental compounds that mimic or impair the action of hormones. EDCs can thus affect a number of physiological and behavioral actions of hormones in animals and humans. Grimaldi et al. compare the effects of EDCs, at the cellular and molecular levels, on human and zebrafish estrogen and peroxisome proliferator-activated γ receptors. They point out that the transcriptional activities of EDCs on human and zebrafish nuclear receptors exhibit marked differences. They conclude that caution should be exercised regarding extrapolation of EDC screening tests in zebrafish models toward hazard assessment for human physiopathology. Rosenfeld focuses on the effect of bisphenol A and phthalate exposure on parental behavior and the impact of developmental exposure to these EDCs on social behavior. They provide convincing evidence that these chemicals alter the organizational and activational programming the brain.

It is our hope that this research topic will become a major set of references for comparative endocrinologists and neurobiologists and will raise the interest of other scientists who are not (yet) involved in this fertile research domain.

## Author Contributions

All authors listed have made a substantial, direct, and intellectual contribution to the work and approved it for publication.

## Conflict of Interest Statement

The authors declare that the research was conducted in the absence of any commercial or financial relationships that could be construed as a potential conflict of interest.
